# Research priorities for oral health in people with severe mental ill-health

**DOI:** 10.1136/bmjment-2026-302673

**Published:** 2026-05-27

**Authors:** Easter Joury, Ellie Heidari, Derek Tracy, Imad Barngkgei, David Shiers, Gordon Johnston, Kamaldeep Bhui

**Affiliations:** 1 Division of Dentistry, School of Medical Sciences, The University of Manchester, Manchester, UK; 2 King’s College London, London, UK; 3 South London and Maudsley NHS Foundation Trust, London, UK; 4 National University of Science and Technology, Nasiriyah, Iraq; 5 Greater Manchester Mental Health NHS Foundation Trust, Manchester, UK; 6 Independent Peer Researcher, Scotland, UK; 7 Department of Psychiatry, University of Oxford, Oxford, UK

**Keywords:** Mood Disorders, Bipolar and Related Disorders, Schizophrenia Spectrum and Other Psychotic Disorders

## Abstract

**Background:**

People with mental disorders bear an excessive burden of oral diseases. This burden can exacerbate the personal, social and economic impacts of mental disorders. There is a need for transformational research to tackle this problem, which should start with co-setting research priorities with people with lived experience and key stakeholders.

**Objective:**

This national Priority Setting Partnership (PSP) aimed to identify the top 10 research priorities for oral health among people with mental disorders.

**Methods:**

Following the James Lind Alliance approach, this three-stage PSP engaged people with mental disorders, carers and staff of all levels and decision-makers from healthcare, social care and non-governmental organisations. In stage 1, questions for research were gathered via an online survey. Summary questions were then formed and checked against existing evidence. In stage 2, unanswered questions were compiled into an online shortlisting survey. In stage 3, a consensus workshop was held to determine the top 10 research priorities.

**Findings:**

From 1214 questions received in stage 1, 60 unanswered questions were formed. Based on 2377 shortlisting survey responses, the 25 top-ranked questions were taken to the consensus workshop, where the top 10 research priorities were determined by 27 participants. The most important research question was ‘What are the best ways to integrate oral health within physical health checks and follow-up care for people with severe mental ill-health?’ Other questions focused on the capability of primary care, mental health and dental teams in supporting oral health, integrated system-level models of dental care and financial incentives for professionals.

**Discussion:**

This PSP identified the top 10 research priorities for oral health among people with mental disorders, which would guide future research and funding aimed at reducing the stark inequalities in oral health between this group and the general population.

**Clinical implications:**

Addressing the priorities identified in this consultation through transformational research would lead to impactful changes in healthcare and public health practice and policy. This in turn would address the burden of oral diseases among people with mental disorders and contribute towards improving their mental and physical health and recovery.

**WHAT IS ALREADY KNOWN ON THIS TOPIC**

- People with mental disorders bear an excessive burden of oral diseases, which aggravates their mental and physical multimorbidity and hinders recovery.
- Despite this importance, this area remains under-researched.

**WHAT THIS STUDY ADDS**

- This is the first consultation in the area of mental health and oral health that co-set research priorities with people with mental disorders, carers, clinicians and decision-makers involved in supporting them, using robust approach.

**HOW THIS STUDY MIGHT AFFECT RESEARCH, PRACTICE OR POLICY**

- Priorities identified here will guide future transformational research and funding aimed at tackling oral health inequalities between this group and the general population.
- This in turn would lead to impactful changes in healthcare and public health practice and policy.

## Introduction

 People with mental disorders bear a substantially higher burden of oral diseases than the general population. For example, based on recent evidence syntheses, people with severe mental illness were three times more likely to have lost all their natural teeth, four times more likely to have periodontal (gum) disease and five times more likely to have tooth decay experience than their counterparts without severe mental illness.^1 2^ They were also twice as likely to have late detection of oral cancer.^3^ Besides this high prevalence, oral diseases can increase the personal, social and economic impacts of mental illness. People with mental disorders have voiced the profound impacts of poor oral health on their quality of life. These include experiencing high levels of pain, disturbing daily functions such as eating, sleeping and socialising and experiencing social stigma and limited employment opportunities.^4^,^7^ Oral diseases were the third most common reason for preventable hospital admissions among people with severe mental illness in the UK.^8^

Mental disorders, physical multimorbidity and oral diseases share the same social determinants and have complex bidirectional interactions that can have synergistic impacts in terms of premature mortality and excessive burden of morbidity.^9 10^ For example, tooth loss, tooth decay and periodontitis, especially in their severe stages, can precipitate and perpetuate mental disorders and physical conditions such as schizophrenia, cardiovascular disease and type 2 diabetes.^11^,^13^ Oral diseases can influence the development and progression of mental disorders through three main mechanisms.^9^ First, they may trigger neuroinflammation via inducing chronic systemic inflammation, direct invasion of oral bacteria or their molecules to the brain and/or communication between oral bacteria or their molecules and brain-resident microglia.^12^ Second, oral diseases can induce chronic psychological stress through causing pain and other profound impacts on sleeping, speaking, eating, socialising and self-esteem. These impacts can be debilitating and hence can be precipitating and perpetuating factors for many mental disorders.^9^ Discrimination and stigma related to poor dental appearance may further compromise job opportunities and social interactions, exacerbating mental health challenges.^14^ Finally, tooth loss can lead to poor nutrition, which may contribute to the onset or worsening of mental disorders.^15^

Despite the above stark oral health inequalities, improving oral health in people with mental disorders has been under-researched. Joury *et al* called in their critical review for actions to address this gap and support future transformational oral health research for this group.^9^ They proposed that these actions should start with setting patient-centred research priorities. Co-setting research priorities with people with lived experience, carers, practitioners and decision-makers is key for fulfilling the principles of ethics and social justice as well as for informing research that will have impact and value for money.^16^ It creates a systematic and transparent approach to involve and assist policy makers and research funders in making investment decisions based on uncertainties that are important and relevant to the needs of end-users.^17^ It could also be used as an advocacy means to bring about change in the priorities of different organisations or groups. Furthermore, the process provides opportunities for mutual learning between different stakeholders and facilitates establishing partnerships that are important for conducting future impactful research.

### Objective

This consultation aimed to conduct a national Priority Setting Partnership (PSP) to identify the top 10 research priorities for oral health and dental care among people with mental disorders.

## Methods

**Figure 1:**
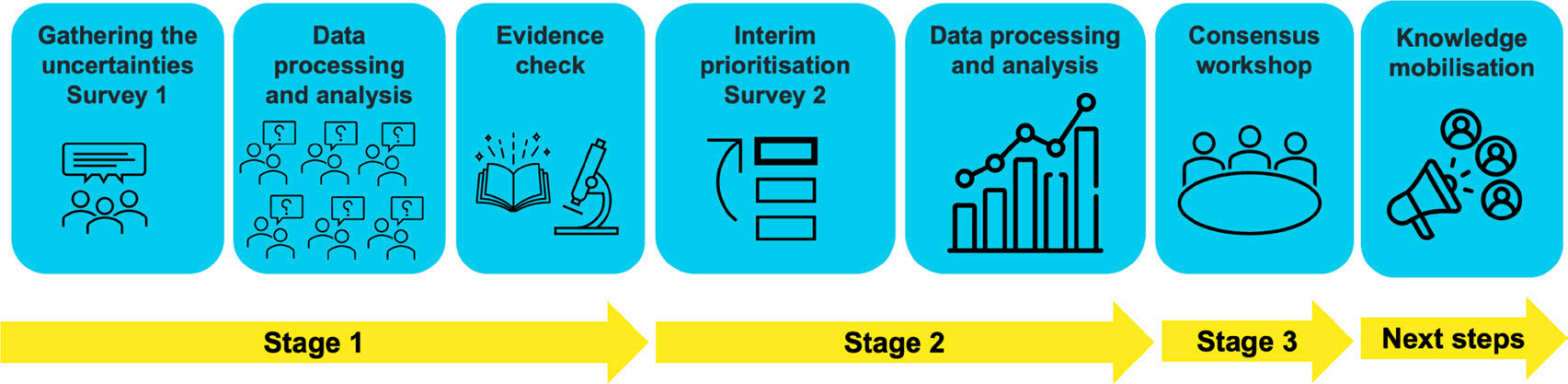
RESTART Priority Setting Partnership approach and process.

Following the James Lind Alliance (JLA) approach,^18^ we set up a Steering Group and carried out this PSP in three stages (figure 1).

### Establishing a Steering Group

We formed a Steering Group that included individuals with mental disorders (n=2), carers (n=2) and mental health (n=2) and dental professionals (n=2) from diverse backgrounds (age, gender, ethnicity, mental disorders experience and health discipline). The Steering Group members brought knowledge of the condition, an understanding of the populations of people with lived experience, carers and clinicians and access to their networks. The Steering Group was responsible for the management and coordination of this PSP and organising its activities, including publicity and overseeing the collation, aggregation and checking of the uncertainties.

### Scope

The scope of the present PSP was focused on oral health and dental care among people with mental disorders, such as those with schizophrenia, bipolar disorder, other psychotic and mood disorders, in the UK. This PSP was named ‘RESTART’, which stands for RESearch prioriTies for orAl health and dental caRe among people with severe menTal ill-health. The phrase ‘severe mental ill-health’ was used instead of ‘mental disorders’ for sensitive language purposes, as suggested by people with lived experience, who considered the former term to be recovery-oriented and less stigmatising. Thus, we hereinafter use ‘severe mental ill-health (SMI)’.

#### Stage 1: gathering the uncertainties

An online survey (OnlineSurvey, JISC, UK) invited people with SMI, carers and relatives and staff of all levels and decision-makers from healthcare, social care and non-governmental organisations that support individuals with SMI to list their top three questions that they would like to be answered. Participant demographic information was collected. This included age, gender, ethnicity, geographic location and their connection to SMI (ie, a person with SMI; a parent, relative, spouse, child or carer of someone with SMI or a professional, individual or decision-maker who works or volunteers for a healthcare, social care or non-governmental organisation that supports individuals with SMI). This information helped check if any group was under-represented among received responses, and hence more engagement was required with them. We also recognised that some people with lived experience might need support from others to respond, and we requested that this be indicated in the corresponding survey question. The survey ran from March 2022 to July 2022. During this time, it was promoted among the above stakeholders. We engaged with various organisations that support individuals with SMI to participate and assist in disseminating the survey further through their newsletters, email circulations, websites, social media platforms and networks. The survey was also promoted at the Right To Smile event and ‘Mind Your Smile’ national live X Chat (#MindYourSmile). Additionally, the lead author presented the RESTART PSP to relevant national and regional Experts by Experience groups, clinical networks and communities of practice. Promotional materials were developed including a short video and social media posts linking to the survey.

##### Forming summary research questions

Out-of-scope submissions were identified and removed. Within-scope duplicate or similar questions were grouped together to form summary research questions. The latter retained the sense of what the respondents meant but rephrased the content to form a clear and researchable question. Two pairs of researchers (EJ/EH and EJ/IB) independently identified out-of-scope and within-scope submissions and thematically analysed them. Any discrepancies in data processing and/or analysis were discussed by each pair, and consensus was reached. All summary research questions were reviewed and agreed on by consensus of the Steering Group. They then underwent an evidence check by the lead author to determine if they had been adequately answered by available literature. A question was considered adequately answered if there was a well-conducted systematic review identifying relevant high-quality evidence and strong recommendations. The evidence search was carried out between January and March 2023 across MEDLINE, EMBASE and the Cochrane Database of Systematic Reviews. Unanswered and partially answered questions were included in the interim prioritisation survey.

### Stage 2: interim prioritisation

A second online survey (Qualtrics, Provo, USA) invited stakeholders to select their top 10 most important summary research questions. This was done in two steps. First, respondents selected their top 10 summary research questions. Second, they ranked these top 10 questions from most to least important. This interim prioritisation survey was shared and promoted in a similar fashion to the first online survey. It ran between June and August 2023.

Questions were assigned scores based on their rankings using a reverse scoring system. For example, the question with ranking 1 (representing the highest rank) was assigned score 10 (the highest score). The individual scores for each question were then added together to obtain an overall score. To ensure equal weight was given to all stakeholder groups regardless of their actual numbers, this scoring process and overall score calculations were carried out separately for each stakeholder group: people with SMI, carers and relatives and professionals and decision-makers. The average of the overall scores across the three stakeholder groups was then calculated for each question to work out the final interim prioritised list. The top 25 ranked summary research questions were included in the consensus workshop.

### Stage 3: consensus workshop

The consensus workshop was held, bringing together stakeholders to determine the final top 10 priorities. Our Steering Group Expert by Experience members advised to run the consensus workshop over two half days to reduce burden on participants with SMI. The workshop was conducted online in two 3-hour sessions in April 2024 and chaired by the lead author. Involvement in the workshop was sought from respondents who indicated interest when participating in the interim prioritisation survey, as well as through the abovementioned Experts by Experience and professional groups and networks. The list of participants was selected, reviewed and agreed by the Steering Group, ensuring balanced representation of all stakeholders and diverse backgrounds. Participants were financially remunerated for their participation (£25 per hour for each participant according to the National Institute for Health and Care Research (NIHR) public contributors’ policy).

Prior to the workshop, all participants were asked to provide short biographical information about themselves, and review and rank the shortlist of 25 questions under consideration. Participants, especially those with lived experience, were encouraged to contact the lead author should they want to have informal conversations to understand more about the workshop process and expectations, and/or to answer any questions or queries they might have. During the workshop, reaching consensus took place in four rounds of discussions over 2 days. To ensure power balance and handling any potential discursive barriers in both small and whole group discussions, facilitators used various techniques. These included establishing ground rules that promote equal participation and reciprocal relationship, as well as underscore the fact that all views are valid and respected. Facilitators used round-robin sharing in small groups, actively listened to and encouraged quieter members to contribute either verbally or in writing via the chat box. They also encouraged participants to stay on topic and gently interrupted if needed to ensure everyone can contribute. Furthermore, facilitators ensured that conversations were jargon free and participants had opportunities to ask for clarification and to comment on the language used. They also took notes of participants’ input to check power balance through cross-referencing contributions from people with lived experience with agreed priorities ranking. Furthermore, as the workshop spanned 2 days, this gave additional reflection time for people with lived experience to formulate their thoughts further and contribute with greater confidence.

Round 1 involved small group discussions and ranking. Participants were split into three small groups (with equal mix of Experts by Experience and professionals to promote diversity of perspectives and robust discussion). Each participant, in turn, contributed their views on the shared list of 25 questions they felt most or least strongly about. These were noted down by the group facilitator, and the process continued until all ideas and views were expressed. Participants were encouraged to seek clarification on the listed 25 questions. The small group moved the discussion to ranking the list of 25 questions. The facilitator and e-technologist organised the questions as e-cards on the screen in three rough groups: those which were thought to be most important, those thought to be least important and those not mentioned or where there was divergence of view. Participants were then invited to start discussing the order of the e-cards, with a view to ranking all of them in order. They were also encouraged to take account of the interim ranking findings presented for each question on the e-card. Round 2 involved whole group review. The workshop chair presented the combined ranking of questions from the three groups. Questions, comments and concerns were discussed with the whole group present.

The following day, groups were reallocated, again ensuring balanced representation. Round 3 involved final small group ranking to discuss and revise the combined ranked list from round 2. The facilitator and e-technologist organised the e-cards on the screen in the combined order. The discussion focused more on the top 15 questions and any other questions that participants felt strongly about. A full ranking was done and recorded. Round 4 involved final whole group review. The workshop chair presented the new combined ranking of questions from the three groups. The ranking was discussed in the large group, with the aim of agreeing the top 10 by the end of the discussion session. The workshop chair encouraged all participants to contribute to the discussion equally. The top 10 questions were agreed. A workshop feedback and evaluation form was sent to participants after the workshop.

### Findings

The findings of the present PSP are summarised across the three stages: stage 1: gathering the uncertainties; stage 2: interim prioritisation and stage 3: consensus workshop.

#### Stage 1: gathering the uncertainties

From the initial survey, there were 593 individuals and organisations, yielding 1214 questions. Respondent demographic information is summarised in table 1. People with SMI constituted most respondents (42%; n=249), followed by professionals and decision-makers (37.3%; n=221) and carers and relatives (20.7%; n=123).

**Table 1:** RESTART Priority Setting Partnership participant demographic information

Characteristics	Gathering the uncertainties (n=593)	Interim prioritisation (n=2887)	Consensus workshop (n=27)
Age (years)			
≤24	49 (8.3)	293 (10.1)	3 (11.1)
25–34	255 (43)	1153 (39.9)	1 (3.7)
35–44	107 (18)	724 (25.1)	6 (22.2)
45–54	90 (15.2)	131 (4.5)	2 (7.4)
55–64	66 (11.1)	56 (1.9)	7 (25.9)
≥65	21 (3.5)	16 (0.6)	1 (3.7)
Missing	5 (0.8)	524 (18.2)	7 (25.9)
Gender			
Female	353 (59.5)	1138 (39.4)	15 (55.6)
Male	221 (37.3)	1158 (40.1)	7 (25.9)
Non-binary/third gender	7 (1.2)	30 (1)	0 (0)
Prefer to self-describe	0 (0)	33 (1.1)	0 (0)
Prefer not to answer	12 (2)	28 (1)	1 (3.7)
Missing	0 (0)	510 (17.7)	4 (14.8)
Ethnicity			
White	480 (80.9)	2084 (72.2)	13 (48.1)
Mixed	19 (3.2)	92 (3.2)	3 (11.1)
Asian	36 (6.1)	104 (3.6)	4 (14.8)
Black	34 (5.7)	53 (1.8)	2 (7.4)
Other	4 (0.7)	5 (0.2)	1 (3.7)
Prefer not to answer	20 (0.3)	49 (1.7)	0 (0)
Missing	0 (0)	510 (17.7)	4 (14.8)
Location			
England	522 (88)	1981 (68.6)	21 (77.8)
Scotland	34 (5.7)	186 (6.4)	1 (3.7)
Wales	22 (3.7)	121 (4.2)	1 (3.7)
Northern Ireland	15 (2.5)	99 (3.4)	0 (0)
Missing	0 (0)	510 (17.7)	4 (14.8)
Stakeholder group			
People with SMI	249 (42)	352 (12.2)	7 (25.9)
Carers/Relatives	123 (20.7)	411 (14.2)	5 (18.5)
Professionals/Decision-makers	221 (37.3)	1624 (56.3)	15 (55.6)
Missing	0 (0)	510 (17.7)	0 (0)

RESTART, RESearch prioriTies for orAl health and dental caRe among people with severe menTal ill-health; SMI, severe mental ill-health.

A total of 60 summary research questions underwent an evidence check. These 60 questions were either unanswered or partially answered. The latter were questions about barriers and enablers to oral health self-care and dental care utilisation among people with SMI. Evidence check identified a few studies including a narrative review that focused on such questions.^19^ Based on the above, all 60 summary questions were included in the prioritisation survey (online supplemental appendix 1).

#### Stage 2: interim prioritisation

The second survey received 2887 responses from individuals and organisations, of which 510 were excluded because respondents did not indicate their connection to SMI, leaving 2377 responses for analysis. Respondent demographic information is summarised in table 1. Most (56.3%; n=1624) respondents were professionals and decision-makers, followed by carers and relatives (14.2%; n=411) and people with SMI (12.2%; n=352). The top 25 highest-ranked combined questions, weighted equally according to stakeholder group, are presented in online supplemental appendix 2.

#### Stage 3: consensus workshop

**Figure 2:**
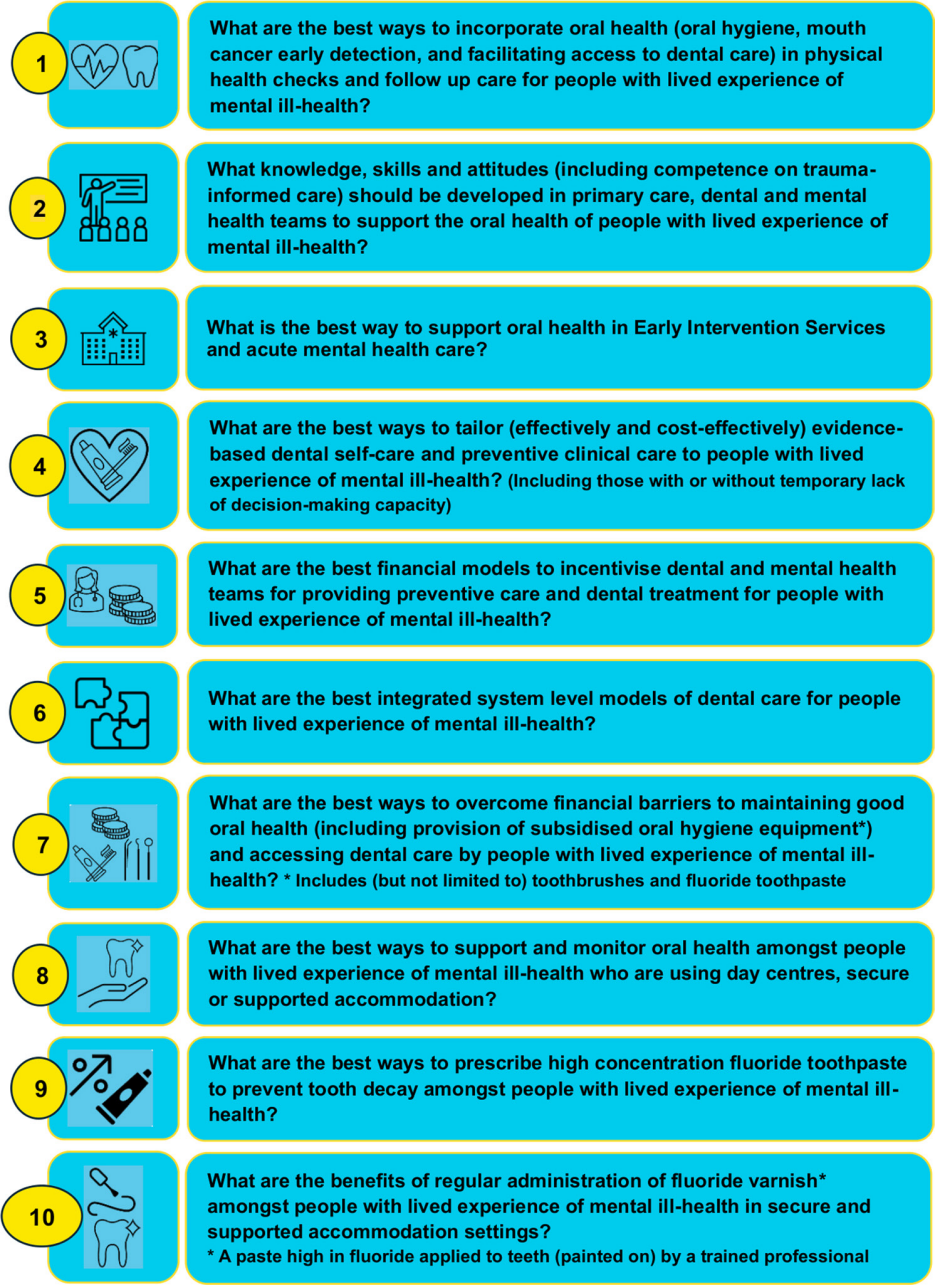
RESTART Priority Setting Partnership top 10 research priorities for oral health and dental care among people with severe mental ill-health.

The consensus workshop was attended by 27 participants, including seven people with SMI, five carers, seven mental health professionals (of different levels), six dental professionals (of different levels) and two policy makers. Participant demographic information is summarised in table 1. Two Steering Group members observed the workshop and three experienced facilitators led the breakout groups. The final top 10 research priorities are presented in figure 2. The most important research question identified was: ‘What are the best ways to integrate oral health (oral hygiene support, mouth cancer early detection and facilitating access to dental care) within physical health checks and follow-up care for people with lived experience of mental ill-health?’ Other key questions focused on the knowledge, skills and attitudes of primary care, dental and mental health teams in supporting oral health, tailoring oral health self-care to people with SMI, integrated system-level models of dental care and financial models to incentivise healthcare professionals. Twenty participants completed the feedback and evaluation form. Findings are summarised in online supplemental appendix 3.

## Discussion

The RESTART PSP identified the top 10 research priorities for oral health and dental care among people with SMI, as determined jointly by stakeholders. To our knowledge, this is the first PSP to address this under-researched area in this vulnerable population.

The strengths of the RESTART PSP include following the JLA approach, which is a standardised, reproducible and transparent process. JLA emphasises equal involvement of stakeholders particularly patients and clinicians. The abovementioned steps taken before and during the workshop to ensure and check power balance between people with lived experience and other stakeholders empowered people with lived experience to join and influence the discussion discourse and priorities selected.^20^ This in turn fostered trust, openness and willingness to collaborate and contribute beyond the present PSP, as highlighted in the evaluation and feedback received (online supplemental appendix 3). More equitable power sharing leads to a higher level of stakeholders’ engagement, ownership and accountability, which in turn can lead to improved quality, relevance and practicality as well as higher uptake, implementation and impact of end outcomes.^16^ Moreover, JLA uses a combination of online surveys and nominal group technique to prioritise research uncertainties. This gives JLA PSP a unique strength compared with other common PSP approaches, such as Delphi surveys, which rely on online surveys only. A further strength of the RESTART PSP is the diverse and high participation across its three stages (a total of 3507 participants).

Despite the above strengths, the RESTART PSP has a number of limitations. The use of JLA approach is time and resource intensive, due to the nature and length of the prioritisation process and stakeholder engagement. Additionally, although the RESTART PSP’s materials were not translated into other languages, targeted engagements with community and voluntary organisations supporting ethnic minority groups were undertaken. A further potential limitation was holding the consensus workshop online. Although online discussions have several advantages, such as removing common barriers associated with accessibility, transportation and travelling, as well as geographical remoteness and dispersion, they are not without limitations.^21 22^ The latter include digital exclusion, limited access to non-verbal cues and less turn-taking in the conversation. These potential limitations of online discussions might affect the group dynamic and impact the quality and representation of the PSP outcomes if they compromised the ability of participants, particularly those with lived experience, to effectively, openly and equally input and contribute to the group discussions and/or to build trust with the facilitator and other participants. Limited access to non-verbal cues might lead to misinterpretation or mask feelings of unease and uncertainty.^21^ The current PSP undertook measures to mitigate such potential limitations. This included facilitating the option to join the online workshop through phone and offering the opportunity for individuals to contact the PSP team should they require help to attend the workshop, or if they want to have informal conversations to answer their questions and queries. A safety protocol was designed and communicated with participants including contacting the lead author should they feel uncomfortable or distressed. Attendance and presence during the workshop were monitored. The abovementioned workshop preparation steps, the small and whole group workshop structure and the facilitation techniques used helped build rapport, foster trust and ensure equal participation by all stakeholders, particularly those with lived experience. Lastly, the current PSP included UK participants only. Nonetheless, the identified research priorities appear to be globally relevant to other high-income countries as well as low- and middle-income countries.^23^

Previous consultations that set oral health research priorities for the general population or specific vulnerable groups are limited in number, rarely involved people with lived experience and were often poorly reported in terms of their approaches and processes.^24^,^26^ Despite this scarcity and heterogeneity, common research priorities were identified across all of these oral health consultations, including the current one. These related to integrated models of care, tailored oral health prevention and self-care support, overcoming financial barriers to oral health and dental access and building the wider workforce competence in oral health for people with SMI, people with additional needs, older people and people with periodontitis and diabetes.^24^,^26^ This is not surprising given that these priorities address key barriers commonly faced by these vulnerable groups, and are often associated with adverse social factors such as deprivation, as well as the management of multiple long-term conditions (MLTC), including oral conditions. Despite these similarities, some differences exist across oral health research priority consultations. For example, priorities related to digital interventions to provide dental care and the development of medications to treat simultaneously periodontitis and diabetes were identified only in previous consultations.^25 26^ Conversely, priorities related to optimal financial models to incentivise dental and non-dental teams to provide oral healthcare, as well as the application of implementation science for specific preventive measures, such as high concentration of fluoride toothpaste and fluoride varnish, were uniquely identified in the present PSP.

Research priorities identified by the RESTART PSP are aligned with current national policy, potentially increasing the likelihood of future research funding. For example, England’s Neighbourhood Health Framework outlines the government’s implementation programme of the county’s 10-Year Health Plan.^27^ The Framework introduced mechanisms to support person-centred, proactive and integrated services, with a strong emphasis on prevention and continuity of care for people with MLTC. This would be enabled through new contracting and payment models, and a rethinking of workforce roles, skills and ways of working across the system. This policy context presents a timely opportunity to support funding research addressing RESTART PSP’s identified priorities that align with these strategic objectives and system enablers. Emerging evidence also suggests the feasibility of such research. For instance, the Restart Smiling study co-created a tailored oral hygiene intervention for people with severe mental illness, integrated within their physical health checks and follow-up care, and delivered by primary care and mental health professionals.^28^ A further example is the feasibility study on a link worker intervention to facilitate access to dental care among people with severe mental illness, including those attending Early Intervention Services.^29^

The consensus workshop, as opposed to the interim prioritisation survey, facilitated a different type and level of engagement with stakeholders. Workshop discussions helped participants contextualise the identified uncertainties, explain why they were prioritised, check the language used and refine questions further and capture complexities. This might explain the changes noted in ranking questions between the interim and final prioritisation. For example, based on the workshop discussions, participants prioritised complex system-level interventions, such as the best ways to integrate oral health into physical health checks and workforce training for people with SMI, rather than more clinically focused interventions, such as the benefits of regular fluoride varnish application in secure and supported accommodation settings.

Next steps include knowledge mobilisation and evaluating the RESTART PSP impact. The Steering Group created a dissemination strategy that includes, besides a peer-reviewed publication and conference presentation, a national dissemination event to share the research priorities with key stakeholders including research funders, commissioners and policy makers. Policy briefings, public-facing summaries, social media posts and press releases of the research priorities will be co-produced. These will also be available on the websites of the partner organisations. Tracking and evaluating the impact of a PSP is challenging.^18^ It can take a significant amount of time to progress from priorities identification to starting funded research, and longer time for that research to report its outcomes and impact. The uptake of the RESTART priorities will be assessed through their incorporation into commissioned research funding calls, projects and agendas.^18 30^ A search in major funders’ databases such as UK Research and Innovation, the NIHR and medical and dental research charities will be undertaken for commissioned calls, awarded grants and funded projects relating to priorities identified by the RESTART PSP. This search will be combined with searching databases such as MEDLINE and EMBASE to identify further research projects and policy documents that referenced the RESTART PSP and/or addressed its identified priorities. Additionally, a search in key governmental and non-governmental organisations’ websites for relevant position or consensus statements as well as guidelines will be carried out. Examples include policy and guidelines documents of the Department of Health and Social Care, the National Institute for Health and Care Excellence, the Royal Colleges of Psychiatrists and General Practitioners and the British Society of Special Care Dentistry.

In conclusion, the RESTART PSP identified the top 10 research priorities for oral health and dental care among people with SMI, as determined jointly by people with SMI, carers, staff of all levels and decision-makers from healthcare, social care and non-governmental organisations. This ensures that future oral health research aligns with real-world needs. The identified priorities would guide future research and funding efforts to address oral health inequalities between this vulnerable group and the general population.

## Supplementary material

10.1136/bmjment-2026-302673 online supplemental appendix 1

## Data Availability

Data are available on reasonable request.
