# Supplementary material for: Research priorities for oral health in people with severe mental ill-health
Source: BMJ Ment Health. 2026 May 27;29(1):e302673. doi: 10.1136/bmjment-2026-302673 (PMC13218139; doi:10.1136/bmjment-2026-302673)
Supplement: online supplemental appendix 1 [file bmjment-29-1-s001.pdf]

Research priorities for oral health in people with severe mental ill-health

Joury E, Heidari E, Tracy D, Barngkgei I, Shiers D, Johnston G, Bhui K

Appendix 1. List of summary research questions included in the interim prioritisation.

1. What are the benefits of providing preventative and treatment dental care for people with severe mental conditions?
2. What are the benefits of providing dental assessment, preventative and treatment dental care to people in the early phase of psychosis?
3. Is using high concentration fluoride toothpaste effective in preventing tooth decay in people with severe mental conditions?
4. What are the benefits of regular administration of fluoride varnish in people with complex psychosis in secure and supported accommodation settings?
5. Is preventative dental care more cost-effective than dental treatments in people with severe mental conditions?
6. Does preventative dental care have less carbon footprint compared to dental treatments in people with severe mental conditions?
7. What are the best ways to screen, early detect and manage oral cancer in people with severe mental conditions?
8. What are the best ways to support and monitor oral health in people with severe mental conditions in day centres, secure and supported accommodation settings?
9. What is the best way to support oral health during a psychosis or other mental health crisis?
10. What knowledge, skills and attitudes should dental teams acquire to provide dental care for people with severe mental conditions, and support their mental and physical health?
11. What is the best skill-mix (staff types) in dental teams to deliver dental care for people with severe mental conditions?
12. What are the best ways to retain and expand the dental workforce capacity (number) and capability (competency) in special care dentistry (of all levels) to cater for the needs of people with severe mental conditions?
13. What are the best financial models to incentivise dental teams for providing preventative dental care and treatment for people with severe mental conditions?

14. What are the best ways to overcome financial barriers to maintaining good oral health and accessing dental care in people with severe mental conditions?

15. Which dental care settings are best suited to provide dental care for people with severe mental conditions?

16. What is the impact of NHS dental contract changes on improving access, quality and continuity of dental care for people with severe mental conditions?

17. How best to commission dental services to provide adequate dental care for people with severe mental conditions?

18. What are the best models of dental care for people with severe mental conditions?

19. What Chronic (dental) Care Model looks like for people with severe mental conditions?

20. What reasonable adjustments of dental services are needed to facilitate access, provision and continuity of quality dental care for people with severe mental conditions?

21. What are the best ways to incorporate oral health in physical health checks, care plan and follow up care for people with severe mental illness?

22. Is providing a one-stop shop facility for delivering physical and dental health checks and follow up care for people with severe mental illness effective?

23. What interventions can be undertaken by non-dental staff working with people with severe mental conditions to support their oral health?

24. What knowledge, skills and attitudes should primary care and mental health teams acquire to support oral health in people with severe mental conditions?

25. What are the best financial models to incentivise primary and mental health teams for supporting oral health in people with severe mental conditions?

26. What knowledge, skills and attitudes should carers acquire to support oral health in people with severe mental conditions?

27. What role can peer support workers play in improving oral self-care and receiving dental care in people with severe mental conditions?

28. What role can community, voluntary and social enterprise sector play in improving oral self-care and receiving dental care in people with severe mental conditions?

29. How best to integrate primary and secondary care, mental health services, dental services, social care and community, voluntary and social enterprise

sector to cater for the mental, physical and oral health needs of people with severe mental conditions?

30. What is the best way to include dental professionals in multidisciplinary teams managing the mental and physical health of people with severe mental conditions?

31. What is the best way to share relevant information on people with severe mental conditions amongst health professionals managing and supporting their mental, physical and oral health?

32. What are the best methods to motivate people with severe mental conditions to look after their teeth?

33. What are the best ways to tailor evidence-based dental preventative recommendations to people with severe mental conditions?

34. What are the best methods to give information on maintaining oral health in people with severe mental conditions?

35. What are the best ways to incorporate oral self-care (oral hygiene and reduced frequency of sugar consumption) within general lifestyle interventions for people with severe mental conditions?

36. Is providing subsidised oral hygiene tools effective in improving oral hygiene in people with severe mental conditions?

37. What is the best way for people with severe mental conditions to self-monitor their oral health?

38. What “wait well” looks like for people with severe mental conditions waiting for dental treatment?

39. Are population-based oral health interventions effective in reducing the burden of oral diseases in people with severe mental conditions?

40. Is integrating oral health within young people mental health services and school-based programs effective in preventing oral diseases later in the life course?

41. What interventions can reduce oral health inequalities “within” people with severe mental conditions?

42. Can oral health deterioration be an early sign of the deterioration of mental health?

43. What is the nature and impact of the relationship between severe mental conditions, physical health conditions and oral diseases?

44. What is the status and three-year trajectory of oral health in people experiencing first episode of psychosis?

45. What are the best ways to prevent or remedy the adverse effects of psychiatric medications?

46. What are the best ways to manage dental phobia and anxiety in people with severe mental conditions?
47. What is the best way to control pain during the provision of dental care for people with severe mental conditions?

48. What are the best interventions to enhance dental treatment adherence in people with severe mental conditions?

49. What are the clinical and patient reported outcome measures of dental care delivery for people with severe mental conditions?

50. What is the success rate of dental care in people with severe mental conditions?

51. Can dental care have harmful effects on people with severe mental conditions?

52. What is the role of Artificial Intelligence in supporting oral health in people with severe mental conditions?

53. Are remote dental consultations safe and effective for people with severe mental conditions?

54. What are the best ways to support digitally excluded people with severe mental conditions to improve their oral self-care and access dental care?

55. What is the best way to support oral health in people with severe mental conditions lacking decision-making capacity?

56. What trauma-informed dental care looks like for people with severe mental conditions?

57. What are the best interventions to support people with severe mental conditions who have previous poor experiences with dental care?

58. What personalised dental care looks like for people with severe mental conditions?

59. What are the best ways to improve communication between dental teams and people with severe mental conditions and their carers?

60. What are the best ways for dental teams to engage and proactively reach out to people with severe mental conditions who are not engaged with dental care?

Appendix 2. List of top 25 questions from the interim prioritisation.

List of questions	Stage 2 interim prioritisation		
	**Service users ranking**	**Carers ranking**	**Professionals ranking**
**1.** What are the best ways to screen, early detect, and manage oral (mouth) cancer in people with severe mental conditions?	4th	1st	2nd
**2.** What are the benefits of regular administration of fluoride varnish (a paste high in fluoride painted on teeth by a dentist or dental professional) in people with psychosis in secure and supported accommodation settings?	3rd	5th	1st
**3.** Is using high concentration fluoride toothpaste effective in preventing tooth decay in people with severe mental conditions?	2nd	3rd	4th
**4.** Is preventative dental care more cost-effective than dental treatments provided for people with severe mental conditions?	7th	2nd	3rd
**5.** Does preventative dental care have a smaller carbon footprint compared to dental treatments provided for people with severe mental conditions?	1st	4th	10th
**6.** What are the best methods to motivate people with severe mental conditions to look after their teeth?	5th	6th	7th
**7.** What are the best ways to tailor evidence-based dental preventative recommendations to people with severe mental conditions?	6th	9th	5th
**8.** What are the best methods to give information on maintaining oral health in people with severe mental conditions?	9th	7th	8th
**9.** What is the best way for people with severe mental conditions to self-monitor their oral health?	8th	11th	6th
**10.** What are the best ways to support and monitor oral health in people with severe mental conditions who are using day centres, secure or supported accommodation?	14th	8th	11th
**11.** What knowledge, skills and attitudes should primary care, dental and mental health teams	11th	14th	9th

acquire to support the oral health of people with severe mental conditions?			
**12.** What are the best ways to overcome financial barriers to maintaining good oral health and accessing dental care by people with severe mental conditions?	10th	13th	13th
**13.** What is the best way to support oral health during a psychosis or other mental health crisis?	12th	12th	14th
**14.** What is the best way to support oral health in people with severe mental conditions lacking decision-making capacity?	18th	10th	12th
**15.** What is the best skill-mix (the range of skills and mix of different types of staff) in mental and dental teams to provide dental care for people with severe mental conditions?	16th	16th	15th
**16.** Is providing subsidised oral hygiene tools effective in improving oral hygiene in people with severe mental conditions?	17th	15th	17th
**17.** What "wait well" looks like for people with severe mental conditions waiting for dental treatment?	13th	17th	22nd
**18.** What is the impact of NHS dental contract changes on improving access, quality and continuity of dental care for people with severe mental conditions?	19th	21st	16th
**19.** What reasonable adjustments of dental services are needed to facilitate access, provision and continuity of quality dental care for people with severe mental conditions?	20th	19th	18th
**20.** What are the best ways to retain and expand the dental workforce number and capability (competency) in special care dentistry (of all levels) to cater for the needs of people with severe mental conditions?	21st	20th	19th
**21.** Which dental care settings are best suited to provide dental care for people with severe mental conditions?	15th	22nd	24th
**22.** What are the best financial models to incentivise dental and mental health teams for providing	23rd	18th	23rd

preventative care and dental treatment for people with severe mental conditions?			
**23.** How best to commission dental services to provide adequate dental care for people with severe mental conditions?	24th	24th	20th
**24.** What are the best models of dental care for people with severe mental conditions?	25th	23rd	21st
**25.** What are the best ways to incorporate oral health in physical health checks, care plan and follow up care for people with severe mental illness?	26th	26th	25th

Appendix 3. RESTART concensus workshop feedback and evaluation (n=20).

Feedback and evaluation questions	Strongly agree	Agree	Neither agree nor disagree	Disagree	Strongly disagree
1. RESTART research priorities are jointly owned, and attendees worked together to achieve a joint understanding.	80%	20%	0%	0%	0%
2. RESTART workshop included people with diverse perspectives and skills to contribute.	80%	20%	0%	0%	0%
3. Everyone was of equal importance, and the knowledge of all was respected and valued.	55%	40%	5%	0%	0%
4. The workshop was accessible, and there have been different ways that have enabled me to contribute to the discussion.	60%	40%	0%	0%	0%
5. There was an emphasis on building and maintaining relationships.	35%	50%	10%	0%	0%
6. I benefited from attending RESTART workshop and working together.	55%	45%	0%	0%	0%
7. The content of RESTART workshop was of high quality.	75%	25%	0%	0%	0%
8. The delivery of RESTART workshop was of high quality.	80%	15%	5%	0%	0%
9. There was enough time for discussion and questions.	30%	45%	15%	10%	0%
10. I would recommend RESTART workshop to others.	75%	25%	0%	0%	0%

11. What impact did participating in the workshop have on you?

- Able to contribute loved experience and learn some of the structures of creating change in mental health.
- To review and map services - so that that patients can be signposted and supported by the PH teams.

- Opportunity that patients with severe mental illness can be supported to access dental care at whatever level is needed.
- I thought the workshop was informative, interesting and I benefited from it.
- It gave me insight from different stakeholders perspective.
- It greatly enhanced my knowledge through exchanging ideas and sharing experiences among multi-skilled groups. It also highlighted the importance of the oral and mental-health link.
- I learnt how important it is for a person mental health through good dental care/practice. The speakers were also very informative.
- I learnt so much on how as a carer I can support a person with MH challenges especially around dental health care and what is available and how important it can be to benefit a person’s wellbeing.
- Considering how Occupational Therapy in mental health can include more focus on oral health.
- The differing perspectives and experiences present widened my knowledge.
- Made me think more about my own oral health and how mental health impact services had an impact of my own oral health (not allowing me to have a toothbrush for about 4 years).
- Often the bridge between academia and clinical dentistry is long, with each group having their own priorities. There is often work and time needed to translate and instigate research into everyday clinical practice. It was refreshing to be invited to draw on my "boots on the ground" experience and input into such a significant and important research project.
- Statistics on inequality was helpful; and understanding the language and culture of the project was enlightening.
- Made me more aware of different views on the subject.
- Better understanding of interprofessional working between service providers and users and the value of everyone's views and contribution in decision making

- Helped me understand the different viewpoint of people from different background and perspective. Gaining knowledge on important factors of mental health and real life situations.

- Learning about national initiatives as well as what's happening in individual Trusts.

- Made me interested in learning more about the work and research being undergone on this topic.

- The importance of providing the necessary and accurate information for people with mental health problem with regards to dental care.

- I found the workshop informative and interesting.

- I found this has a direct impact on considering how I see and approach some of our patients in special care.

12. If there is anything else you would like to add, please do so here:

    - Thank you for the day.

    - Thank you for hosting and facilitating a great project.

    - Many thanks for all great efforts put into this important workshop.

    - I would love to continue to participate in future workshops.
    - I look forward to participating in future elements of the project. Many thanks for the opportunity.

    - Thank you for the opportunity to participate throughout an extremely interesting and rewarding process.

    - Refresher training is required.

    - Thank you for this opportunity and if there are more (either to volunteer or else) I would love to take part.

    - I thoroughly enjoyed this workshop and would absolutely love to contribute more.

Additional feedback via email

- Thank you for a very stimulating discussion yesterday. Looking forward for today.
- That’s brilliant Easter you’ve captured everything. Thank you for Looking at the language too.
- Many thanks for your great efforts in the very well-organised workshop for today. Very much looking forward to taking part in finalising the ranking tomorrow!
- Thank you for leading today's and yesterday's workshops so skilfully and thoughtfully. It was a privilege to be involved in such an inclusive piece of work.
- I wanted to write and say what an absolutely fantastic workshop and it was absolutely fascinating. It was a real delight and thank you ever so much. In the next stages if I can be of any help please do let me know.
- I really enjoyed the small group session and getting to have a discussion with the other participants. Best wishes for the rest of the project.
